# The Role of HLA Antigens and Steroid Dose on the Course of COVID-19 of Patients After Kidney Transplantation

**DOI:** 10.3389/fmed.2021.730156

**Published:** 2021-11-01

**Authors:** Ivana Dedinská, Petra Skálová, Karol Graňák, Matej Vnučák, Tatiana Baltesová, Zuzana Žilinská, Miloš Jeseňák

**Affiliations:** ^1^Jessenius Faculty of Medicine, Transplantation Center, University Hospital, Comenius University, Martin, Slovakia; ^2^Transplant Department, L. Pasteur's University Hospital, Košice, Slovakia; ^3^Department of Urology, Medical Faculty, Renal Transplantation Center, University Hospital, Comenius University, Bratislava, Slovakia; ^4^Department of Pediatrics, Department of Pneumology and Phthisiology, Department of Clinical Immunology and Allergology, Jessenius Faculty of Medicine, University Hospital, Comenius University, Martin, Slovakia

**Keywords:** kidney transplantation, COVID-19, HLA class I and class II typing, steroid dose, immunosuppression

## Abstract

**Background:** Kidney transplant recipients appear to be at higher risk for critical COVID-19. Our analysis aimed to identify the possible risk factors for a severe course of the COVID-19 disease and to determine the influence of selected human leukocyte antigens (HLAs) on the course of the disease.

**Methods:** This is a retrospective, multicenter analysis that included patients that were confirmed to be severe acute respiratory syndrome coronavirus-2 (SARS-CoV-2) positive after kidney transplantation (KT). The group of patients was divided into two subgroups according to the course of the infection, as follows: non-hospitalized and hospitalized.

**Results:** A total of 186 patients (men, 69.4%) with confirmed SARS-CoV-2 positivity were included in the group. The following independent risk factors for the outcome of hospitalization were identified: the age at the time of infection [odds ratio (OR) = 1.19, *P* < 0.0001], a body mass index (BMI) >29.9 kg/m^2^ (OR = 7.21, *P* < 0.0001), <7.5-mg prednisone dose/day (OR = 2.29, *P* = 0.0008), and HLA-DQ2 with a protective nature (OR = 0.05, *P* = 0.0034).

**Conclusions:** Higher doses of corticosteroids (>7.5 mg/kg) in standard immunosuppressive regimes and HLA-DQ2 appear to be protective factors in our analysis.

## Introduction

The novel coronavirus, severe acute respiratory syndrome coronavirus-2 (SARS-CoV-2), was first identified toward the end of 2019 in Wuhan, in the Hubei province of China, and spread rapidly throughout the world. The clinical symptoms of the disease vary from a completely asymptomatic course through fever, cough, the development of bronchopneumonia, up to multiorgan failure (1).

Kidney transplant recipients appear to have a particularly high risk for critical COVID-19 illness due to chronic immunosuppression and coexisting conditions (2). In comparison with the general population, patients who underwent kidney transplantation (KT) from diabetes mellitus, arterial hypertension, or cardiovascular diseases suffer more often. Immunosuppressive treatment is also a precondition for the worse course of the disease, most frequently with the combination of calcineurin inhibitors (CNI), mycophenolate mofetil (MMF), and corticosteroids (2–4).

Several analyses compared the course of COVID-19 infection in patients after KT to the non-transplanted population for their similarities, with large inter-individual differences. According to data from the European Renal Association COVID-19 Database register (ERACODA), the 28-day probability of death in 1,073 patients (28% after KT and 72% in a chronic dialysis program) was 21.3% in the patients who had kidney transplants and 25% in those undergoing dialysis (5). The available data from China show the high mortality of patients with end-stage kidney disease (33%) (6), and the data from Spain and Italy have shown the 30% mortality rate of dialyzed patients (7, 8). Known publications from New York reported a 16–30% mortality rate in the group of patients after KT (2, 9, 10).

The risk factors for hospitalization or death in this population remain to be similar to the general population (11). Death is more common among elderly patients and those with pre-existing pulmonary disease (11, 12).

The treatment of COVID-19 infection in patients who had kidney transplants does not significantly differ from the treatment of the non-transplanted population; however, the question remains whether and when it is necessary to discontinue immunosuppression. There are several protocols for the immunosuppressive management of transplanted patients with COVID-19, which reflect either the clinical progress of the disease, laboratory findings, or a combination of both (13–16). The data concerning the usage of hydroxychloroquine, remdesivir, or tocilizumab in this specific group of patients are more controversial than promising (17–19). The recently published data of using bamlanivimab for patients after solid organ transplantation are promising for the treatment of patients with mild-to-moderate COVID-19 who are at high risk of progression to hospitalization (20).

The human leukocyte antigen (HLA) is a critical component of the viral antigen presentation pathway in humans, and the HLA gene locus plays a fundamental role in human adaptive immunity. The genetic variability across the HLA alleles is known to be associated with outcomes in many different diseases and could be a key determinant in the susceptibility and severity of COVID-19. The HLA type influences the T cell-mediated response to viral infections and is therefore implicated in the morbidity and mortality of a SARS-CoV-2 infection (21, 22). Several smaller studies have indicated a possible association of the HLAs of group 1 (A1 and A2) with a more severe course of infection (23–25).

Our analysis aimed to identify the risk factors for a severe course (hospitalization) of COVID-19 disease in a group of patients after KT and the risk factors for COVID-19 fatalities, with a focus on the parameters before the infection (not on the parameters worsening infection during its course). Another aim was to determine the influence of the selected HLAs on the course of the disease.

## Materials and Methods

This was a retrospective, multicenter cohort analysis that included patients with kidney transplants who had positive test results for SARS-CoV-2 monitored in the Slovak Republic (transplantation centers Martin, Košice, and Bratislava) from March 2020 to December 2020. The positivity was confirmed by a real-time polymerase chain reaction (RT-PCR) test. We recorded the age at the time of test positivity, the time (months) from KT, a diabetes mellitus and arterial hypertension case history, and the body mass index (BMI) of all the patients. We evaluated the type and doses/levels of immunosuppression [tacrolimus, MMF, mycophenolic acid (MPA), and corticosteroids], HLA (A, B, DR, and DQ), and finally, the development of post-COVID syndromes. Post-COVID syndrome has been defined as the persistence of certain clinical difficulties at least 4 weeks after the infection has passed (i.e., fatigue, shortness of breath, insomnia, memory problems, “brain fog.” heart palpitation, depression, and/or anxiety). The fatal outcome was defined as death in direct association with COVID-19, with respiratory failure confirmed by autopsy. In the patients who died, we recorded the time—the duration of illness (confirmed by positive test results) up to the time of death.

Inclusion criteria: Age >18 years, positive test results for SARS-CoV-2 by PCR
Exclusion criteria: Age <18 years, failed to follow-up, antirejection treatment in the last 6 months.

**Figure 1:**
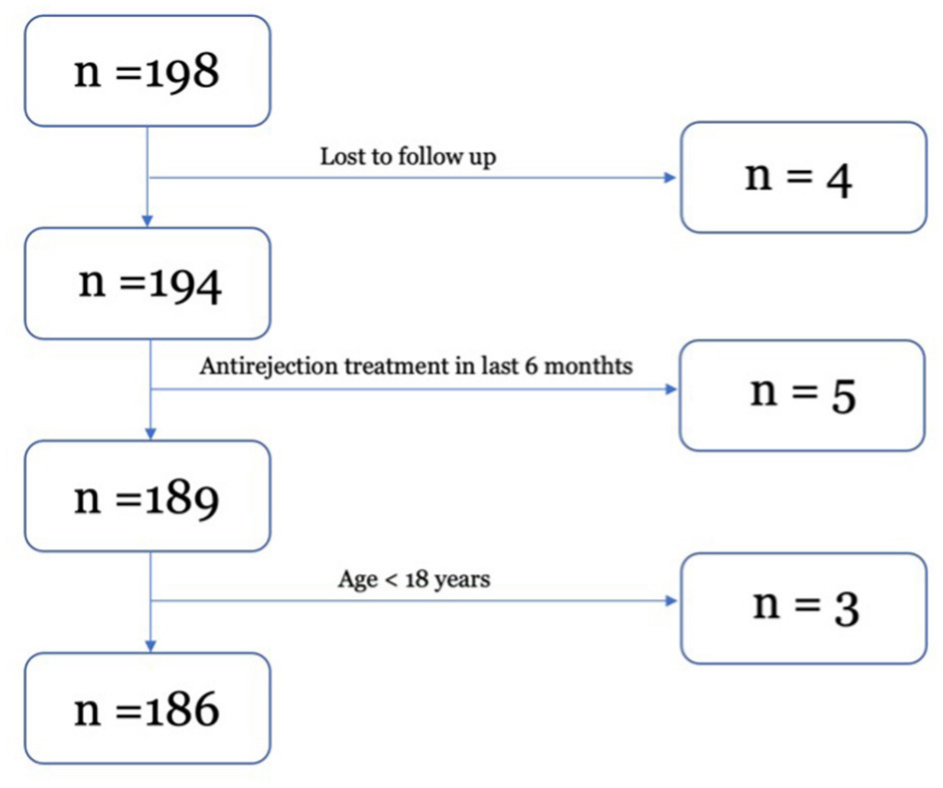
Flowchart of the study.

The flowchart of the study is shown in Figure 1.

### Immunosuppression

The maintenance immunosuppression consisted of tacrolimus and MPA (1,080-mg daily dose until the second week after transplantation, followed by a 720-mg daily dose, or adjusted individually according to the immunology risk of the patient). Furthermore, 500 mg of methylprednisolone was administered intravenously (IV) on day 0 and day 1, followed by the administration of 20 mg of prednisone until the 2nd week after transplantation, 15 mg of prednisone until the 4th week after transplantation, 10 mg of prednisone until the 12th week after transplantation, and 7.5 mg of prednisone until the 12th month after transplantation, with 5 mg of prednisone administered daily thereafter. The tacrolimus target levels were according to the protocols used in the Slovak Republic; 10–15 ng/ml 1–3 months after transplantation, followed by 5–10 ng/ml 3–6 months after transplantation, and 3–6 ng/ml for patients >6 months after transplantation.

The group of patients was divided into two subgroups according to the course of infection [based on the Centers For Disease Control (CDC) and Prevention Therapeutic Management of Adults With COVID-19 Last Updated: February 11, 2021], as follows:

- Subgroup 1: Patients not requiring hospitalization (asymptomatic, oligosymptomatic, or medium–severe course). Asymptomatic patients with maintained immunosuppression in the treatment regimen. Patients with a medium–severe course of COVID-19 had reduced immunosuppression [discontinuation of MPA or MMF, reduction of (CNI) by 50%].
- Subgroup 2: Severe course. Hospitalized patients with completely discontinued immunosuppression were administered 6 mg of dexamethasone IV for 24 h for a minimum of 10 days added to the treatment.

### HLA Typing

The HLA typing of all patients was performed with PCR-based procedures using a sequence-specific primer (SSP). After the performance of the PCR, the amplified DNA fragments were size-separated using agarose gel electrophoresis and then visualized, documented, and interpreted.

### Statistical Analysis

We used a certified statistical program, MedCalc version 13.1.2 (VAT registration no. BE 0809 344 640, Member of International Association of Statistical Computing, Ostend, Belgium), to perform statistical analyses. The continuous data were compared using Student's *t*-test, and a Wilcoxon rank-sum test was used for the analysis of the time after KT as it is non-parametric data. An χ^2^-test and Fisher's exact test were used for categorical variables.

Univariable and multivariable logistic regressions were used to assess the monitored parameters to predict the risk of hospitalization and death. The statistically significant parameters assessed in the univariable analysis were entered into the multivariable model adjusted for the time after KT, sex, tacrolimus level, and MMF. The cut-off level for Prednison dose used in the logistic regression models was based on the average dose of Prednison in our group of patients.

A Kaplan–Meier survival analysis was used for the comparison of the survival rates of the hospitalized and non-hospitalized patients. We considered *P* < 0.05 to be statistically significant.

### Ethical Approval

All procedures involving human participants have been approved according to the ethical standards of the institutional research committee, including the 1964 Helsinki Declaration and its later amendments of comparable ethical standards. The informed consent of the included participants was checked and approved by the ethical committees of the University hospital and the Jessenius Faculty of Medicine, and all signed informed consents are to be archived for at least 20 years after the research was completed.

The clinical and research activities being reported are consistent with the Principles of the Declaration of Istanbul as outlined in the Declaration of Istanbul on Organ Trafficking and Transplant Tourism.

## Results

A total of 186 patients (men, 69.4%) with confirmed SARS-CoV-2 positivity were included in the group.

The group of patients was further divided according to the course of the disease into two subgroups. The criteria for the non-hospitalized patients were met by 147 patients. There were 39 patients who required hospitalization (subgroup 2), as displayed in Table 1.

**Table 1:** Basic group characteristics according to course of infection.

**Characteristic**	**Non-hospitalized *n* = 147**	**Hospitalized *n* = 39**	***P-*value**
Gender—men (%)	67.3	76.9	0.2491
Age at the time of infection (in years)	51.7 ± 10.1	62 ± 11.2	** <0.0001**
Time after KT (months) (mean ± standard deviation)	78.7 ± 54.1 (median, 70)	104.9 ± 63.6 (median, 94)	**0.0104**
BMI (kg/m^2^) (mean ± standard deviation)	28.8 ± 6	29.4 ±7.3	0.5971
Diabetes mellitus (%)	42.9	61.5	**0.0390**
Arterial hypertension (%)	91.8	100	0.0651
Tacrolimus in treatment (%)	91.8	92.3	0.9192
Average TAC level (ng/ml)	4.1 ± 1	4.1 ± 1	1.0000
MPA/MMF in treatment (%)	85.7	84.6	0.8628
Average MPA dose/day (mg)	668 ± 360	720 ± 311	0.4111
Average dose of corticosteroids/day (mg)	7.6 ± 2.9	5.9 ± 1.9	**0.0007**
Post COVID (%)	16.3	46.2	**0.0001**
HLA-A1 (%)	18.4	8	0.1183
HLA-A2 (%)	32	36	0.6372
HLA-A3 (%)	17	24	0.3179
HLA-B8 (%)	14.3	0	**0.0001**
HLA-B35 (%)	12.9	17.4	0.4706
HLA-B12 (%)	12.9	4.3	0.1295
HLA-DR1 (%)	9.5	13.3	0.4888
HLA-DR3 (%)	9.5	10	0.9251
HLA-DR11 (%)	10.9	10	0.8720
HLA-DR52 (%)	17	13.3	0.5784
HLA-DQ2 (%)	19	5.9	**0.0493**
HLA-DQ3 (%)	32	35.3	0.6969
HLA-DQ5 (%)	22.4	23.5	0.8843
HLA-DQ6 (%)	19	29.4	0.1587
Fatalities (%)	2 (*n* = 3)	15.4 (*n* = 6)	**0.0005**

*KT, kidney transplantation; BMI, body mass index; TAC, tacrolimus; MPA, mycophenolic acid; MMF, mycophenolate mofetil; HLA, human leukocyte antigen*.

*The bold values mean statistical significant values*.

At the time of the infection, all the monitored patients received prednisone as a part of their treatment; the patients in subgroup 2 received, on average, a lower dose of prednisone/day, and it was logically associated with the fact that they represented the group with the longer average duration of time after transplantation. The dosing of MPA and MMF was comparable in the monitored subgroups, as well as the average level of tacrolimus. As compared with subgroup 1, the patients in subgroup 2 had a higher age and a higher proportion of patients with diabetes mellitus. Three patients died in subgroup 1 and six patients died in subgroup 2. The post-COVID-19 difficulties of the group are shown in Table 2. Post COVID-19 syndrome was recorded more often in the hospitalized patients, with the most common symptom being fatigue.

**Table 2:** Post-COVID difficulties.

***n* = 42**	
Fatigue, *n* = 21 (%)	50
Shortness of breath, *n* = 5 (%)	11.9
Insomnia, *n* = 3 (%)	7.1
Memory problems, *n* = 2 (%)	4.8
Brain fog, *n* = 2 (%)	4.8
Heart palpitation, *n* = 7 (%)	16.7
Depression and/or anxiety, *n* = 2 (%)	4.8

We used a univariable analysis to determine the risk factors for the outcomes of hospitalization and death, as exhibited in Table 3.

**Table 3:** Univariable analysis (log regression).

**Characteristic**	**Outcome of hospitalization OR (95% CI)**	***P*-value**	**Outcome of death OR (95% CI)**	***P*-value**
Gender—men	1.61 (0.71–3.67)	0.2517	0.40 (0.07–2.20)	0.2961
Age at the time of infection (in years)	1.11 (1.06–1.16)	**<** **0.0001**	1.36 (1.12–1.64)	**0.0013**
Time after KT <12 months	1.27 (0.32–4.96)	0.7233	0.64 (0.03–13.06)	0.7778
BMI >29.9 kg/m^2^	5.62 (2.60–12.12)	**<** **0.0001**	3.36 (0.76–14.82)	0.1089
Diabetes mellitus	2.13 (1.03–4.39)	**0.0400**	2.37 (0.54–10.38)	0.2523
Arterial hypertension	7.28 (0.42–125.84)	0.1718	1.54 (0.07–30.99)	0.7778
Tacrolimus in treatment	1.06 (0.28–3.98)	0.9235	1.27 (0.29–5.62)	0.7457
Average TAC-value >6 ng/ml	0.68 (0.22–2.12)	0.5139	1.35 (0.13–13.09)	0.7957
MPA in treatment	0.91 (0.34–2.45)	0.8625	3.57 (0.19–66.74)	0.3937
Average MPA dose >720 mg/day	1.07 (0.39–2.90)	0.8923	0.27 (0.01–5.22)	0.3937
Average prednisone dose ≤ 7.5 mg/day	4.66 (2.16–9.94)	**0.0001**	6.87 (1.23–38.31)	**0.0278**
HLA-A1	0.39 (0.08–1.83)	0.2334	0.44 (0.02–8.39)	0.5888
HLA-A2	1.20 (0.47–3.06)	0.6944	5.36 (3.02–9.52)	**0.0066**
HLA-A3	1.53 (0.52–4.49)	0.4324	2.2 (0.19–25.47)	0.5280
HLA-B8	0.03 (0.002–0.59)	**0.0207**	0.53 (0.02–10.10)	0.6791
HLA-B35	1.36 (0.39–4.71)	0.6194	1.22 (0.13–11.24)	0.8566
HLA-B12	0.61 (0.12–2.98)	0.5500	0.49 (0.02–9.28)	0.6390
HLA-DR1	1.46 (0.43–4.89)	0.5385	3.19 (0.58–17.33)	0.1791
HLA-DR3	1.05 (0.27–4.00)	0.9366	1.36 (0.15–11.93)	0.7771
HLA-DR11	0.51 (0.24–3.40)	0.8917	0.44 (0.02–7.99)	0.5805
HLA-DR52	0.76 (0.24–2.43)	0.6554	0.28 (0.01–5.09)	0.3934
HLA-DQ2	0.05 (0.002–0.95)	**0.0462**	0.57 (0.02–11.21)	0.7155
HLA-DQ3	1.24 (0.41–3.70)	0.6915	0.25 (0.03–2.14)	0.2098
HLA-DQ5	1.04 (0.30–3.56)	0.9425	7.5 (4.21–13.52)	**0.0033**
HLA-DQ6	1.74 (0.54–5.60)	0.3533	0.50 (0.05–2.48)	0.5322

*OR, odds ratio; CI, confidence interval; KT, kidney transplantation; BMI, body mass index; TAC, tacrolimus; MPA, mycophenolic acid; HLA, human leukocyte antigen*.

*The bold values mean statistical significant values*.

We identified the following risk factors for hospitalization in the monitored group: age at the time of infection, a BMI >29.9 kg/m^2^, diabetes mellitus, and a <7.5-mg dose of prednisone/day. On the other hand, HLA-B8 and -DQ2 appeared to be protective factors. The age at the time of infection and a <7.5-mg dose of prednisone/day were confirmed as risk factors for death. We identified the HLA-A2 and -DQ5 of the HLA antigens as risk antigens.

The application of a multivariable analysis (logistic regression) adjusted for the time after KT, sex, tacrolimus level, and MMF dose confirmed the following as independent risk factors for hospitalization: age at the time of infection, a BMI > 29.9 kg/m^2^, a <7.5-mg prednisone dose/day, and HLA-DQ2 with a protective nature (Table 4). The only independent risk factor for the fatal outcome was the age at the time of infection (Supplementary Table 1).

**Table 4:** Multivariable analysis (log regression), outcome of hospitalization (adjusted for time after KT, sex, tacrolimus level, and MMF).

**Characteristic**	**Outcome of hospitalization OR (95% CI)**	***P*-value**
Age at the time of infection (in years)	1.19(1.11–1.29)	** <0.0001**
BMI >29.9 kg/m^2^	7.21 (1.33–8.94)	** <0.0001**
Diabetes mellitus	1.59 (0.50–5.05)	0.4305
Average prednisone dose ≤ 7.5 mg/day	2.29 (1.02–3.88)	**0.0008**
HLA-B8	2.08 (1.94–3.40)	0.9982
HLA-DQ2	0.05 (0.007–0.37)	**0.0034**

*OR, odds ratio; CI, confidence interval; BMI, body mass index; HLA, human leukocyte antigen*.

*The bold values mean statistical significant values*.

In the end, we compared the patients with a daily dose of steroids of <7.5 and >7.5 mg (Table 5). We confirmed that patients with a lower dose of steroids were significantly hospitalized more often. Seven patients died in this group in comparison with two patients in the group with a higher dose of steroids.

**Table 5:** Basic group characteristics according to steroid dose/day.

**Characteristic**	**Subgroup 1 *n* = 111**	**Subgroup 2 *n* = 75**	***P*-value**
Gender—men (%)	70.3	68	0.7392
Age at the time of infection (in years)	51.8 ± 9.4	54.1 ± 15.6	0.2116
Time after KT (months) (mean ± standard deviation)	118.1 ± 67.8	64.3 ± 39.7	** <0.0001**
BMI (kg/m^2^) (mean ± standard deviation)	28.8 ± 4.9	28.1 ± 7.6	0.4459
Diabetes mellitus (%)	43.2	52	0.2393
Arterial hypertension (%)	91.9	96	0.2653
Tacrolimus in treatment (%)	100	80	** <0.0001**
Average TAC level (ng/ml)	4.0 ± 1.1	4.1 ± 1	0.5291
MPA/MMF in treatment (%)	94.6	73.3	** <0.0001**
Average MPA dose/day (mg)	576 ± 262	864 ± 420	** <0.0001**
Post COVID (%)	24.3	20	0.4925
Hospitalization (%)	36	10.7	**0.0001**
Fatalities (%)	6.3	2.7	0.2634

*KT, kidney transplantation; BMI, body mass index; TAC, tacrolimus; MPA, mycophenolic acid; MMF, mycophenolate mofetil. Subgroup 1: steroid dose <7.5 mg/day. Subgroup 2: steroid dose >7.5 mg/day*.

*The bold values mean statistical significant values*.

**Figure 2:**
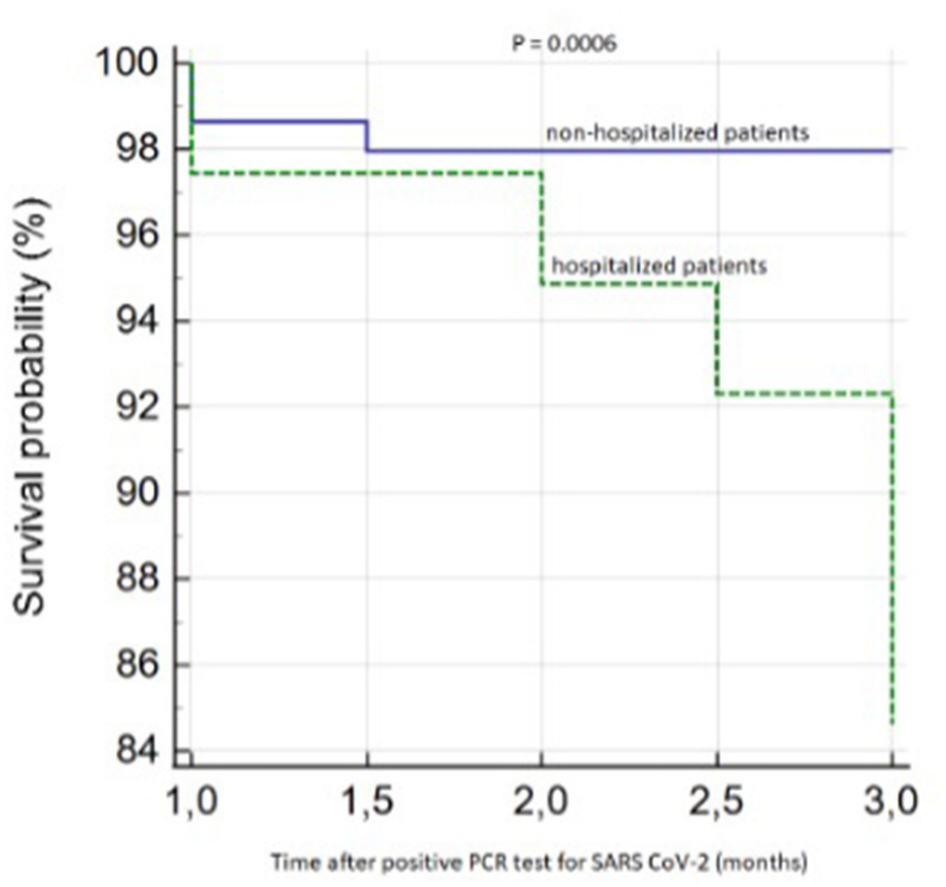
Kaplan–Meier survival analysis (hospitalized vs. non-hospitalized patients).

We confirmed the significantly worse survival in the group of hospitalized patients, as shown in Figure 2.

## Discussion

Our analysis aimed to identify the risk factors for hospitalization due to COVID-19 infection in patients after KT.

Our analysis confirmed that, similar to the general population, the risk group for a severe course of COVID-19 infection and for death is the age of the patient exceeding 59 years. Our data correspond with the conclusions of the ERACODA register, in which 28-day mortality was primarily associated with advanced age in kidney transplant patients (5). An analysis by French authors comparing the course of COVID-19 infection in patients after KT with a non-transplanted group also confirmed a significantly worse course of infection in the group of transplanted patients over 60 years of age. However, compared with the general population, the transplanted patients with a severe course of infection were younger (26).

It is not surprising that a higher BMI (>29.9 kg/m^2^) was found to be a risk factor for hospitalization in patients with COVID-19. Obesity and diabetes mellitus were repeatedly confirmed as independent risk factors for a symptomatic course of COVID-19 in the group of patients after KT (27, 28).

The protective factor of the higher prednisone doses in the normal immunosuppression regime regarding the course of COVID-19 in the population of patients who underwent KT has not yet been described. Dexamethasone, the effect of which was confirmed in the RECOVERY study (29), is normally added to the treatment of all patients hospitalized with COVID-19. Glucocorticoids have been widely used in syndromes closely related to COVID-19 (30–34). However, the data that would support the assumption of the “preventative” effect of corticosteroids on the course of COVID-19 in patients after KT are missing. Patients after KT have generally been administered relatively low doses of prednisone in permanent immunosuppression regimes (from 20 mg in the early post-transplantation period to 2.5–5 mg/day in the late post-transplantation period).

In our group of patients, the dose of prednisone with <7.5 mg/day was a risk factor for hospitalization. The patients in subgroup 2 (those that required hospitalization) were administered with the lower average dose of prednisone (5.9 mg/day). The dosing of corticosteroids is gradually lower based on the time after transplantation; therefore, the patients with the lowest dose of corticosteroids were the patients with the longest duration of time after transplantation.

A great number of disease-protective and disease-susceptible HLA alleles have been well characterized in several viral infections (35). However, little data describe certain HLAs in relation to SARS-CoV-2. A group of Spanish authors assessed HLA class I in relation to the course of COVID-19 in the general population. In a group of 45 patients, they observed the higher values of SARS-CoV-2-binding peptides in the case of HLA-A2, but without any obvious correlation in the clinical picture (36). Another small-scale analysis performed on 62 patients described an association between HLA-A11 and higher mortality (37). In our group of patients in the univariable analysis, we recorded the occurrence of HLA-A2 as a risk factor for death; however, the multivariable analysis did not confirm this finding. On the contrary, a British analysis of 80 patients identified HLA-A2 as a possible protective factor for SARS-CoV-2 infection (38).

In our patient group, the presence of HLA-DQ2 appears to be a protective factor in the case of the need for hospitalization. The complex role of HLA-DQ2 on immune system reactivity is a matter of discussion. Certain studies support its potential in the promotion of interferon (IFN) production. However, other studies showed its negative impact on virus clearance (39). In a study conducted by Benlyamani et al. in critically ill patients, the results indicated the downregulation of HLA-DR molecules in the circulating monocytes (40). In the case of HLA-DQ, data regarding the incidence are available, but not regarding the development of the disease. Some data indicate that HLA-DQ1 can be associated with a higher incidence of COVID-19 (41). It is also important to note that, in a group of almost 2,000 patients from Italy and Spain, the correlation of HLA alleles with the incidence of COVID-19 was not proven (42).

According to the CDC, the most common post-COVID symptoms in the general population are fatigue, shortness of breath, or cognitive problems. The risk factors for post-COVID syndromes that have been identified include the need for hospitalization or being in the intensive care unit (ICU) (43–45). There were 42 patients with post-COVID-19 syndrome identified in our group of patients, and almost 50% of the patients from those who needed hospitalization developed post-COVID syndrome. The most common symptom was fatigue. At present, there is a lack of data about post-COVID syndromes in patients after KT and it needs further investigation.

The limitation of our analysis is the absence of data regarding the treatment and blood tests of patients during hospitalization because the patients were not hospitalized in one COVID center; therefore, the data about treatment would not be homogeneous. On the other hand, this analysis deals with the risk factors for hospitalization for COVID-19 before the onset of the infection. It is also the only analysis that identified certain protective/risk HLA antigens in patients after KT with COVID-19.

## Conclusion

Patients after KT are high-risk patients for a severe course of COVID-19 infection. The obese and older patients (>59 years) can be considered as the group with the highest risks. The higher doses of corticosteroids in standard immunosuppressive regimes (>7.5 mg/kg) and HLA-DQ2 appear to be protective factors. However, these results should be supported with further data on larger samples of patients.

## Data Availability Statement

The raw data supporting the conclusions of this article will be made available by the authors, without undue reservation.

## Ethics Statement

The studies involving human participants were reviewed and approved by University Hospital's and Jessenius Faculty of Medicine's Ethical Committees. The patients/participants provided their written informed consent to participate in this study.

## Author Contributions

ID participated in writing the paper, performance of the research, and data analysis. PS, KG, MV, TB, and ZŽ participated in data analysis. MJ participated in the research design and writing of the paper. All authors contributed to the article and approved the submitted version.

## Conflict of Interest

The authors declare that the research was conducted in the absence of any commercial or financial relationships that could be construed as a potential conflict of interest.

## Publisher's Note

All claims expressed in this article are solely those of the authors and do not necessarily represent those of their affiliated organizations, or those of the publisher, the editors and the reviewers. Any product that may be evaluated in this article, or claim that may be made by its manufacturer, is not guaranteed or endorsed by the publisher.

## Abbreviations

BMI: body mass index
CDC: Centers for Disease Control and Prevention
ERACODA: The European Renal Association COVID-19 Database
HLA: human leukocyte antigen
ICU: intensive care unit
KT: kidney transplantation
MMF: mycophenolate mofetil
MPA: mycophenolic acid
PCR: polymerase chain reaction
TAC: tacrolimus
SARS-CoV-2: severe acute respiratory syndrome coronavirus-2.

## References

[1] Ashish A Yakabu I Winstead R Gowda M Gupta G. COVID-19 in kidney transplantation: epidemiology, management considerations, and the impact on kidney transplant practice. Transplant Direct. (2020) 6:e582. 10.1097/TXD.0000000000001031 33134506 PMC7581117

[2] Akalin E Azzi Y Bartash R Seethamraju H Parides M Hemmige V. Covid-19 and kidney transplantation. N Engl J Med. (2020) 382:2475–7. 10.1056/NEJMc2011117 32329975 PMC7200055

[3] Pereira MR Mohan S Cohen DJ Husain SA Dube GK Ratner LE. COVID-19 in solid organ transplant recipients: initial report from the US epicenter. Am J Transplant. (2020) 20:1800–8. 10.1111/ajt.15941 32330343 PMC7264777

[4] Banerjee D Popoola J Shah S. Ster ICh, Quan V, Phanish M. COVID-19 infection in kidney transplant recipients. Kidney Int. (2020) 97:1076–82. 10.1016/j.kint.2020.03.018 32354637 PMC7142878

[5] Hilbrands LB Duivenvoorden R Vart P Franssen CFM Hemmelder MH Jager KJ. COVID-19-related mortality in kidney transplant and dialysis patients: results of the ERACODA collaboration. Nephrol Dial Transplant. (2020) 35:1973–83. 10.1093/ndt/gfaa261 33151337 PMC7665620

[6] Xiong F Tang H Liu L Tu C Tian JB Lei ChT. Clinical characteristics of and medical interventions for COVID-19 in hemodialysis patients in Wuhan, China. J Am Soc Nephrol. (2020) 31:1387–97. 10.1681/ASN.2020030354 32385130 PMC7350995

[7] Alberici F Delbarba E Manenti C Econimo L Valerio F Pola A. A report from the Brescia Renal COVID Task Force on the clinical characteristics and short-term outcome of hemodialysis patients with SARS-CoV-2 infection. Kidney Int. (2020) 98:20–6. 10.1016/j.kint.2020.04.030 32437768 PMC7206428

[8] Goicoechea M Sánchez Cámara LA Macías N de Morales AM Rojas AG Bascuñana A. COVID-19: clinical course and outcomes of 36 maintenance hemodialysis patients from a single center in Spain. Kidney Int. (2020) 98:27–34. 10.1016/j.kint.2020.04.031 32437770 PMC7211728

[9] Columbia University Kidney Transplant Program. Early description of coronavirus 2019 disease in kidney transplant recipients in New York. J Am Soc Nephrol. (2020) 31:1150–1156. 10.1681/ASN.2020030375 32317402 PMC7269361

[10] Nair V Jandovitz N Hirsch JS Nair Gm Abate M Bhaskaran M. COVID-19 in kidney transplant recipients. Am J Transplant. (2020) 20:1819–25. 10.1111/ajt.15967 32351040 PMC7267603

[11] Favà A Cucchiari D Montero N Toapanta N Centellas FJ Vila-Santandreu A. Clinical characteristics and risk factors for severe COVID-19 in hospitalized kidney transplant recipients: a multicentric cohort study. Amer J Transplant. (2020) 20:3030–41. 10.1111/ajt.16246 32777153 PMC7436908

[12] Oto OA Ozturk S Turgutalp K Arici M Alpay N Merhametsiz O. Predicting the outcome of COVID-19 infection in kidney transplant recipients. BMC Nephrol. (2021) 22:100. 10.1186/s12882-021-02299-w 33740915 PMC7977489

[13] Willicombe M Thomas D McAdoo S. COVID-19 and calcineurin inhibitors: should they get left out in the storm? J Am Soc Nephrol. (2020) 31:1145–6. 10.1681/ASN.2020030348 32312797 PMC7269341

[14] Zhong Z Zhang Q Xia H Wang A Liang W Zhou W. Clinical characteristics and immunosuppressant management of coronavirus disease 2019 in solid organ transplant recipients. Am J Transplant. (2020) 20:1916–21. 10.1111/ajt.15928 32282986 PMC7262295

[15] Meziyerh S Zwart TC van Etten RW Janson JA van Gelder T Alwayn IPJ. Severe COVID-19 in a renal transplant recipient: a focus on pharmacokinetics. Am J Transplant. (2020) 20:1896–901. 10.1111/ajt.15943 32337790 PMC7267503

[16] S. Marx D Moulin B Fafi-Kremer S Benotmane I Gautier G Perrin P. First case of COVID-19 in a kidney transplant recipient treated with belatacept. Am J Transplant. (2020) 20:1944–16. 10.1111/ajt.15919 32282977 PMC7262359

[17] Tang W Cao Z Han M Wang Z Chen J Sun W. Hydroxychloroquine in patients with mainly mild to moderate coronavirus disease 2019: open label, randomised controlled trial. BMJ. (2020) 369:m1849. 10.1136/bmj.m1849 32409561 PMC7221473

[18] Johnson KM Belfer JJ Peterson GR Boelkins MR Dumkow LE. Managing COVID-19 in renal transplant recipients: a review of recent literature and case supporting corticosteroid-sparing immunosuppression. Pharmacotherapy. (2020) 40:517–24. 10.1002/phar.2410 32339304 PMC7267490

[19] Adamsick ML Gandhi RG Bidell MR Elshaboury RH Bhattacharyya RP Kin AY. Remdesivir in patients with acute or chronic kidney disease and COVID-19. J Am Soc Nephrol. (2020) 31:1384–6. 10.1681/ASN.2020050589 32513665 PMC7351006

[20] Dhand A Lobo SA Wolfe K Feola N Nabors C. Bamlanivimab for treatment of COVID-19 in solid organ transplant recipients: early single-center experience. Clin Transplant. (2021) 35:e14245. 10.1111/ctr.14245 33595145 PMC7995073

[21] Yu K He J Wu Y Xie B Liu X Wei B. Dysregulated adaptive immune response contributes to severe COVID-19. Cell Res. (2020) 30:814–6. 10.1038/s41422-020-0391-9 32759967 PMC7403569

[22] Crux NB Elahi S. Human Leukocyte Antigen (HLA) and immune regulation: how do classical and non-classical HLA alleles modulate immune response to human immunodeficiency virus and hepatitis C virus infections? Front Immunol. (2017) 8:832. 10.3389/fimmu.2017.00832 28769934 PMC5513977

[23] Pisanti S Deelen J Gallina AM Caputo M Citro M Abate M. Correlation of the two most frequent HLA haplotypes in the Italian population to the differential regional incidence of Covid-19. J Transl Med. (2020) 18:352. 10.1186/s12967-020-02515-5 32933522 PMC7491019

[24] Shkurnikov M Nersisyan S Jankevic T Galatenko A Gordeev I Vechorko V. Association of HLA class I genotypes with age at death of COVID-19 patients. Front Immunol. (2021) 12:641900. 10.3389/fimmu.2021.641900 33732261 PMC7959787

[25] Wang W Zhang W Zhang J He J Zhu F. Distribution of HLA allele frequencies in 82 Chinese individuals with coronavirus disease-2019 (COVID-19). HLA. (2020) 96:194–6. 10.1111/tan.13941 32424945 PMC7276866

[26] Caillard S Chavarot N Francois H Matignon M Grete C Kamar N. Is COVID-19 infection more severe in kidney transplant recipients? Am J Transplant. (2021) 21:1295–303. 10.1111/ajt.16424 33259686 PMC7753418

[27] Klang E Kassin G Soffer S Freeman R Levin MA Reich DL. Severe obesity as an independent risk factor for COVID-19 mortality in hospitalized patients younger than 50. Obesity. (2020) 28:1595–9. 10.1002/oby.22913 32445512 PMC7283736

[28] Elias M Pievani D Randoux C Louis K Denis B Delion A. COVID-19 Infection in kidney transplant recipients: disease incidence and clinical outcomes. J Am Soc Nephrol. (2020) 31:2413–23. 10.1681/ASN.2020050639 32847984 PMC7609004

[29] RECOVERY Collaborative Group Horby P Lim WS Emberson JR Mafham M Bell JL Linsell L. Dexamethasone in hospitalized patients with covid-19. N Engl J Med. (2021) 384:693–704. 10.1056/NEJMoa2021436 32678530 PMC7383595

[30] Ruan Q Yang K Wang W Jiang L Song J. Clinical predictors of mortality due to COVID-19 based on an analysis of data of 150 patients from Wuhan, China. Intensive Care Med. (2020) 46:846–8. 10.1007/s00134-020-05991-x 32125452 PMC7080116

[31] Stockman LJ Bellamy R Garner P. SARS: systematic review of treatment effects. PLoS Med. (2006) 3:e343. 10.1371/journal.pmed.0030343 16968120 PMC1564166

[32] Arabi YM Mandourah Y Al-Hameed F Sindi AA Almekhlafi GAA Hussein MA. Corticosteroid therapy for critically ill patients with Middle East respiratory syndrome. Am J Respir Crit Care Med. (2018) 197:757–67. 10.1164/rccm.201706-1172OC 29161116

[33] Lansbury LE Rodrigo C Leonardi-Bee J Nguyen-Van-Tam J Lim WS. Corticosteroids as adjunctive therapy in the treatment of influenza: an updated Cochrane systematic review and meta-analysis. Crit Care Med. (2020) 48:e98–106. 10.1097/CCM.0000000000004093 31939808

[34] Siemieniuk RA Meade MO Alonso-Coello P Briel M Evaniew N Prasad M. Corticosteroid therapy for patients hospitalized with community-acquired pneumonia: a systematic review and meta-analysis. Ann Intern Med. (2015) 163:519–28. 10.7326/M15-0715 26258555

[35] Blackwell JM Jamieson SE Burgner D HLA. and infectious diseases. Clin Microbiol Rev. (2009) 22:370–85. 10.1128/CMR.00048-08 19366919 PMC2668228

[36] Iturrieta-Zuazo I Rita CG García-Soidán A de Malet Pintos-Fonseca A Alonso-Alarcón N. Pariente-Rodríguez. Possible role of HLA class-I genotype in SARS-CoV-2 infection and progression: a pilot study in a cohort of covid-19 Spanish patients. Clin Immunol. (2020) 219:108572. 10.1016/j.clim.2020.108572 32810602 PMC7428760

[37] Lorente L Martín MM Franco A Barrios Y Cáceres JJ Solé-Violán J. HLA genetic polymorphisms and prognosis of patients with COVID-19 (2020). Med Intens. (2020) 45:96–103. 10.1016/j.medin.2020.08.004 38620408 PMC7474921

[38] Poulton K Wright P Hughes P Savic S Smith MW Guiver M. A role for human leucocyte antigens in the susceptibility to SARS-Cov-2 infection observed in transplant patients. Int J Immunogenet. (2020) 47:324–8. 10.1111/iji.12505 32623831 PMC7361549

[39] Knierman MD Lannan MB Spindler LJ McMillian CL Konrad RJ Siegel RW. The human leukocyte antigen class II immunopeptidome of the SARS-CoV-2 spike glycoprotein. Cell Rep. (2020) 33:108454. 10.1016/j.celrep.2020.108454 33220791 PMC7664343

[40] Benlyamani I Venet F Coudereau R Gossez M Monneret G. Monocyte HLA-DR measurement by flow cytometry in COVID-19 patients: an interim review. Cytometry A. (2020) 97:1217–21. 10.1002/cyto.a.24249 33125816

[41] Raslan MA Alshahawey M Shehata EM Sabri NA. Does human leukocyte antigen gene polymorphism affect management of COVID-19 patients? Sci J Genet Gene Ther. (2020). 6:1–3. 10.17352/sjggt.000018

[42] Ellinghaus D Degenhardt F Bujanda L Buti M Albillos A Invernizzi P. genomewide association study of severe covid-19 with respiratory failure. N Engl J Med. (2020) 383:1522–34. 10.1056/NEJMoa2020283 32558485 PMC7315890

[43] CDC Post-COVID Conditions. Avaialble online at: https://www.cdc.gov/coronavirus/2019-ncov/long-term-effects.html (accessed July 12, 2021).

[44] Dennis A Wamil M Alberts J Oben J Cuthbertson DJ Wootton D. Multiorgan impairment in low-risk individuals with post-COVID-19 syndrome: a prospective, community-based study. BMJ Open. (2021) 11:e048391. 10.1136/bmjopen-2020-048391 33785495 PMC8727683

[45] Morin L Savale L Pham T Colle R. Figueiredo Sl Harroi A. Writing committee for the COMEBAC study group four-month clinical status of a cohort of patients after hospitalization for COVID-19. JAMA. (2021) 325:1525–34. 10.1001/jama.2021.3331 33729425 PMC7970386

