# Supplementary material for: The Role of HLA Antigens and Steroid Dose on the Course of COVID-19 of Patients After Kidney Transplantation
Source: Front Med (Lausanne). 2021 Nov 1;8:730156. doi: 10.3389/fmed.2021.730156 (PMC8591240; doi:10.3389/fmed.2021.730156)
Supplement: Supplementary file 1 [file Table_1.docx]

**Suppl 1** Multivariable analysis (log regression), outcome of death (adjusted for time after KT, sex, tacrolimus level and MMF)

| **Characteristic** | **Outcome of death**  **OR (95% CI)** | **P-value** |
| --- | --- | --- |
| **Age at the time of infection (in years)** | 1.38 (1.09–1.75) | **0.0063** |
| **Average prednisone dose ≤ 7.5 mg/day** | 0.17 (0.01–0.96) | 0.3260 |
| **HLA-A2** | 1.67 (1.35–4.09) | 0.9990 |
| **HLA-DQ5** | 1.80 (1.22–2.78) | 0.9988 |

Abbreviations: OR, odds ratio; CI, confidence interval; BMI, body mass index; HLA, human leukocyte antigen
